# Identification of cholesterol-assimilating actinomycetes strain and application of statistical modeling approaches for improvement of cholesterol oxidase production by *Streptomyces anulatus* strain NEAE-94

**DOI:** 10.1186/s12866-020-01775-x

**Published:** 2020-04-10

**Authors:** Noura El-Ahmady El-Naggar, Nancy M. El-Shweihy

**Affiliations:** Department of Bioprocess Development, Genetic Engineering and Biotechnology Research Institute, City for Scientific Research and Technological Applications, Alexandria, Egypt

**Keywords:** *Streptomyces* sp. strain NEAE-94, Identification, 16S rRNA, Plackett-Burman design, Box Behnken design

## Abstract

**Background:**

Cholesterol oxidase biosensors have been used to determine the level of cholesterol in different serum and food samples. Due to a wide range of industrial and clinical applications of microbial cholesterol oxidase, isolation and identification of a new microbial source (s) of cholesterol oxidase are very important.

**Results:**

The local isolate *Streptomyces* sp. strain NEAE-94 is a promising source of cholesterol oxidase. It was identified based on cultural, morphological and physiological characteristics; in addition to the 16S rRNA sequence. The sequencing product had been deposited in the GenBank database under the accession number KC354803. Cholesterol oxidase production by *Streptomyces anulatus* strain NEAE-94 in shake flasks was optimized using surface response methodology. The different process parameters were first screened using a Plackett-Burman design and the parameters with significant effects on the production of cholesterol oxidase were identified. Out of the 15 factors screened, agitation speed, cholesterol and yeast extract concentrations had the most significant positive effects on the production of cholesterol oxidase. The optimal levels of these variables and the effects of their mutual interactions on cholesterol oxidase production were determined using Box-Behnken design. Cholesterol oxidase production by *Streptomyces anulatus* strain NEAE-94 was 11.03, 27.31 U/mL after Plackett-Burman Design and Box-Behnken design; respectively, with a fold of increase of 6.06 times compared to the production before applying the Plackett-Burman design (4.51 U/mL).

**Conclusions:**

Maximum cholesterol oxidase activity was obtained at the following fermentation conditions: g/L (cholesterol 4, yeast extract 5, NaCl 0.5, K_2_HPO_4_ 1, FeSO_4_.7H_2_O 0.01, MgSO_4_.7H_2_O 0.5), pH 7, inoculum size 4% (v/v), temperature 37°C, agitation speed of 150 rpm, medium volume 50 mL and incubation time 5 days.

## Background

Cholesterol oxidase (EC 1.1.3.6) is a flavin adenine dinucleotide (FAD)-dependent enzyme that catalyzes cholesterol oxidation to cholestenone (4-cholesten-3-one) and hydrogen peroxide [1]. Cholesterol oxidase of microbial origin exhibits a wide range of industrial applications besides to its clinical applications in order to determine food and serum cholesterol levels which are important in the diagnosis of cardiovascular disease, atherosclerosis and other lipid disorders [2, 3]. It plays an essential role in macrophages and leukocytes lysis [4]. In addition, cholesterol oxidase also involved in the manifestation of viral (HIV) disease, bacterial disease (tuberculosis) and Alzheimer’s disease [5]. In addition, cholesterol oxidase from *Streptomyces natalensis* is required for the biosynthesis of the polyene macrolide pimaricin (antifungal antibiotic) which is used as a mould inhibitor in the food industry [6] to prevent food contamination. It is also used as an antibiotic effective in the treatment of keratitis because it interacts with the molecules of sterols present in fungal cell membranes which cause membrane disruption and leading to intracellular components leakage [7].

The *Bordetella* sp. cholesterol oxidase is used as a promising therapy for lung cancer treatment (both in vitro and in vivo) and led to irreversible cell apoptosis even after addition of cholesterol [8]. *Rhodococcus equi* infects young horses as well as human immuno-compromised patients [9]. Cholesterol oxidase can be used to treat the bacterial infections of *Rhodococcus equi* for the host cells [10]. Purified bacterial cholesterol oxidase showed strong insecticidal activity against boll weevil larvae “*Anthonomus grandis*”, which decreases the cotton yields [11]. When cholesterol oxidase is ingested by cotton boll weevil larvae, larvae die and the fertility of adult females decreases [12]. Cholesterol oxidase also exhibits insecticidal effect when ingested by pink bollworm (*Pectinophora gossypiella*), tobacco budworm (*Heliothis virescens*) and corn earworm (*Helicoverpa zea*) [13].

The intracellular or extracellular cholesterol oxidase produced by a variety of microorganisms, including *Streptomyces natalensis* [6] *Streptomyces cavourensis* [14], *Mycobacterium tuberculosis* [9], *Chromobacterium* sp. DS1 [15], *Bacillus pumilus* W1 and *Serratia marcescens* W8 [16]. In case of pathogenic bacteria, cholesterol oxidases serve as membrane destroying agents and thus contribute to their pathogenicity as a virulence factor [9]. Non-pathogenic bacteria use cholesterol for growth as a carbon and energy sources.

Many scientists have sought to increase the yield of cholesterol oxidase because of low production by several microorganisms. The production of cholesterol oxidase is greatly influenced by the soluble component of the medium and culture conditions, including temperature, incubation time, pH, inoculum size and the rate of agitation [17]. Response surface methodology (RSM) is an effective tool by which the optimum conditions can be determined for a multivariate system. Statistical methods were more effective than the classical method (one-variable-at-a-time) in determining the optimal concentrations of medium components used to produce cholesterol oxidase. Statistical methods offer a range of advantages, including lower experimental numbers, suitability for multi-factor experiments, studying the mutual interactions among the variables, and identifying the most appropriate conditions for maximum enzyme production [18]. Statistical optimization by surface response methodology involves two steps, firstly, the screening of significant factors and, secondly, the optimization of such factors [19].

The objectives of this study were to identify robust cholesterol-assimilating actinomycetes strain with high cholesterol oxidase activity, and to optimize the fermentation conditions for enhanced production of cholesterol oxidase by *Streptomyces anulatus* strain NEAE-94 using response surface methodology.

## Results

**Fig. 1:**
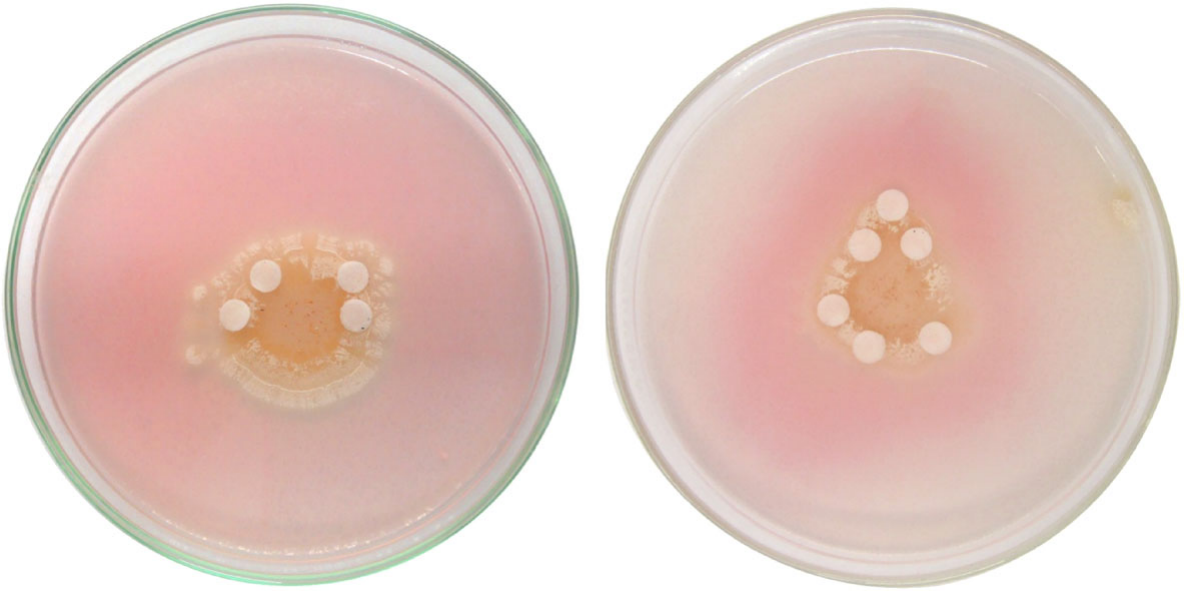
Cholesterol oxidase production of *Streptomyces* sp. NEAE-94 detected by plate assay method

Actinomycete strain, *Streptomyces* sp. strain NEAE-94, has been tested for its cholesterol oxidase activity with a plate-based method, the formation of the pink areas around the colonies indicated the presence of cholesterol oxidase activity (Fig. 1). The process of cholesterol-oxidation is the oxidation of cholesterol with the use of cholesterol oxidase to 4-cholesten-3-one and hydrogen peroxide. The hydrogen peroxide generated by cholesterol oxidation was then combined with 4-aminoantipyrine and phenol by peroxidase to produce quinoneimine coloration (Supplementary Figure 1). The promising strain was identified based on morphological, cultural, physiological and chemotaxonomic characteristics, in addition to 16S rRNA sequence.

### Cultural characteristics of *Streptomyces* sp. strain NEAE-94

**Fig. 2:**
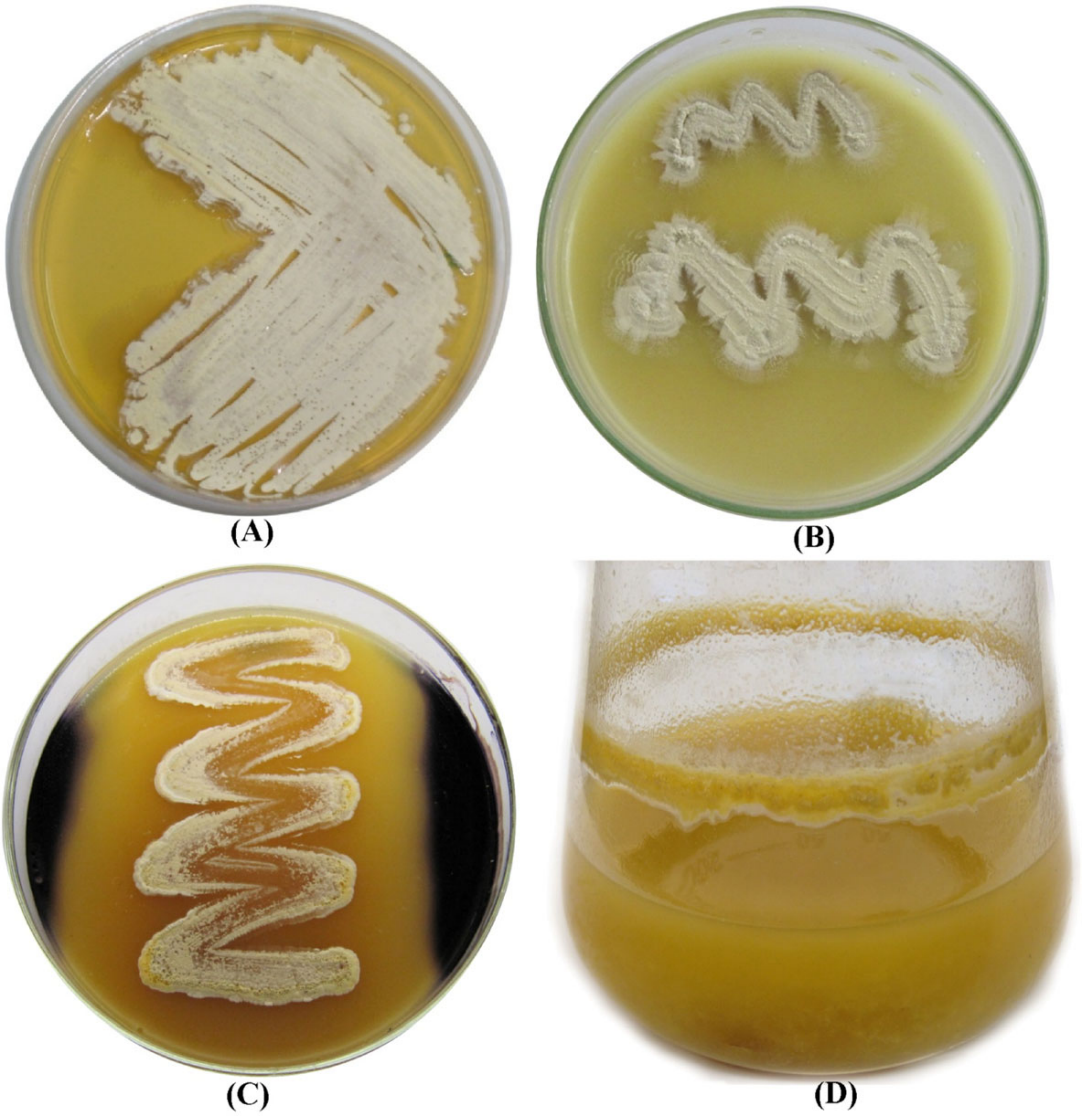
The colored photograph of *Streptomyces* sp. NEAE-94 aerial mycelium after growth on yeast extract -malt extract agar (**a**) and inorganic salt-starch agar media (**b**) at 30°C for 7 days, (**c**) plate assay showing starch hydrolysis by *Streptomyces* sp. strain NEAE-94 and (**d**) cholesterol oxidase production in submerged fermentation

The isolate showed well growth on four different media, including oatmeal agar, inorganic salt-starch agar, tyrosine agar and yeast malt extract agar. Weak growth was observed on glycerol asparagines agar and peptone-yeast extract iron agar (Supplementary Table 1). Aerial mycelium color was whitish yellow on yeast extract-malt extract agar (Fig. 2a); yellow on oatmeal agar, tyrosine agar and inorganic salt-starch agar (Fig. 2b), while is faint yellow on peptone-yeast extract iron agar and glycerol asparagines agar. However, the substrate mycelium develop a yellow color on oatmeal agar, inorganic salt-starch agar, yeast extract -malt extract agar; brownish orange color on tyrosine agar media. Whereas, a faint orange color was developed on both glycerol asparagine agar and peptone-yeast extract iron agar media. A yellow diffusible pigment was produced in inorganic salt-starch agar, yeast extract-malt extract agar, tyrosine agar and glycerol asparagine agar; a faint yellow pigment was produced in oatmeal agar. The diffusible pigment was not pH indicator. No pigments were produced in peptone-yeast extract iron agar.

### Physiological properties of *Streptomyces* sp. strain NEAE-94

The physiological properties of *Streptomyces* sp. strain NEAE-94 are listed in Table 1. It utilized trehalose, D(+)xylose, D(+)mannose, rhamnose, D(+)galactose, raffinose, D(−)fructose, D(+)glucose, L-arabinose, sucrose, maltose and cellulose, but could not utilized ribose as the sole carbon source. *Streptomyces* sp. strain NEAE-94 has reduced nitrate to nitrite. Coagulation and peptonization of milk were positive. Production of protease, α–amylase (Fig. 2c), cellulase, gelatinase and asparaginase was positive. On the other hand, production of uricase, lecithinase and chitosanase was negative. The optimal growth temperature was 30°C and the optimal pH was 7.0. *Streptomyces* sp. strain NEAE-94 grew in the presence of NaCl up to 5% (w/v). *Streptomyces* sp. strain NEAE-94 showed positive antimicrobial activities against *Staphylococcus aureus, E. coli, Bacillus subtilis* and *Pseudomonas aeruginosa*, but no activities were shown against *Candida albicans*, *Rhizoctonia solani*, *Aspergillus niger, Fusarium oxysporum*, *Alternaria solani*, *Sacchromyces cerevisiae*, *Bipolaris oryzae* or *Klebsiella pneumonia*. *Streptomyces* sp. strain NEAE-94 did not produce melanoid pigments in tyrosine agar, peptone-yeast extract iron agar or tryptone-yeast extract broth.

**Table 1:** Phenotypic properties of *Streptomyces* sp. strain NEAE-94 and related *Streptomyces* species. Reference species properties have been taken from Bergey’s Manual of Systematic Bacteriology [20]

Characteristic	***Streptomyces*** sp. strain NEAE-94	***Streptomyces anulatus***	***Streptomyces flavofuscus***	***Streptomyces fimicarius***	***Streptomyces parvus***
Aerial mycelium on ISP medium 2	Whitish -yellow	Yellow or white	Yellow, green-yellow	Yellow or white	Yellow
Diffusible pigment	Yellow	Trace of yellow	Yellow-gray-brown		Yellow
Substrate mycelium on ISP medium 2	Yellow	Pale yellow; grayish yellow or yellowish brown	Yellow- gray-brown	Not distinctive	Not distinctive
Surface of spores	Smooth	Smooth	Smooth	Smooth	Smooth
Morphology of spore chain	*Rectiflexibles*	*Rectiflexibiles*	*Rectiflexibiles*	*Rectiflexibiles*	*Rectiflexibiles*
Melanin production on Tyrosine agar, peptone-yeast extract iron agar or tryptone-yeast extract broth	–	–	–	–	–
**Utilization of carbon sources**					
D(+) glucose, D(−) fructose, L-arabinose, D(+) xylose, rhamnose	+	+		+	+
Sucrose, raffinose	+	+		±	±
D(+) mannose, cellulose	+	+		+	
Trehalose, D(+) galactose, maltose	+				
Ribose	–				

Abbreviations: *+* Positive, *−* Negative, *±* Doubtful, Blank cells no data available. L-asparaginase, gelatinase, cellulose, protease and α–amylase were produced by *Streptomyces* sp. strain NEAE-94 while chitosanase, lecithinase and uricase were not produced. Maximum NaCl tolerance (5%, w/v).The optimal growth temperature was 30°C and optimal pH was 7.0. It exhibited positive antimicrobial activities against *Staphylococcus aureus, E. coli, Bacillus subtilis* and *Pseudomonas aeruginosa*. Nitrate reduction, coagulation and peptonization of milk were positive

### Morphological features of *Streptomyces* sp. strain NEAE-94

**Fig. 3:**
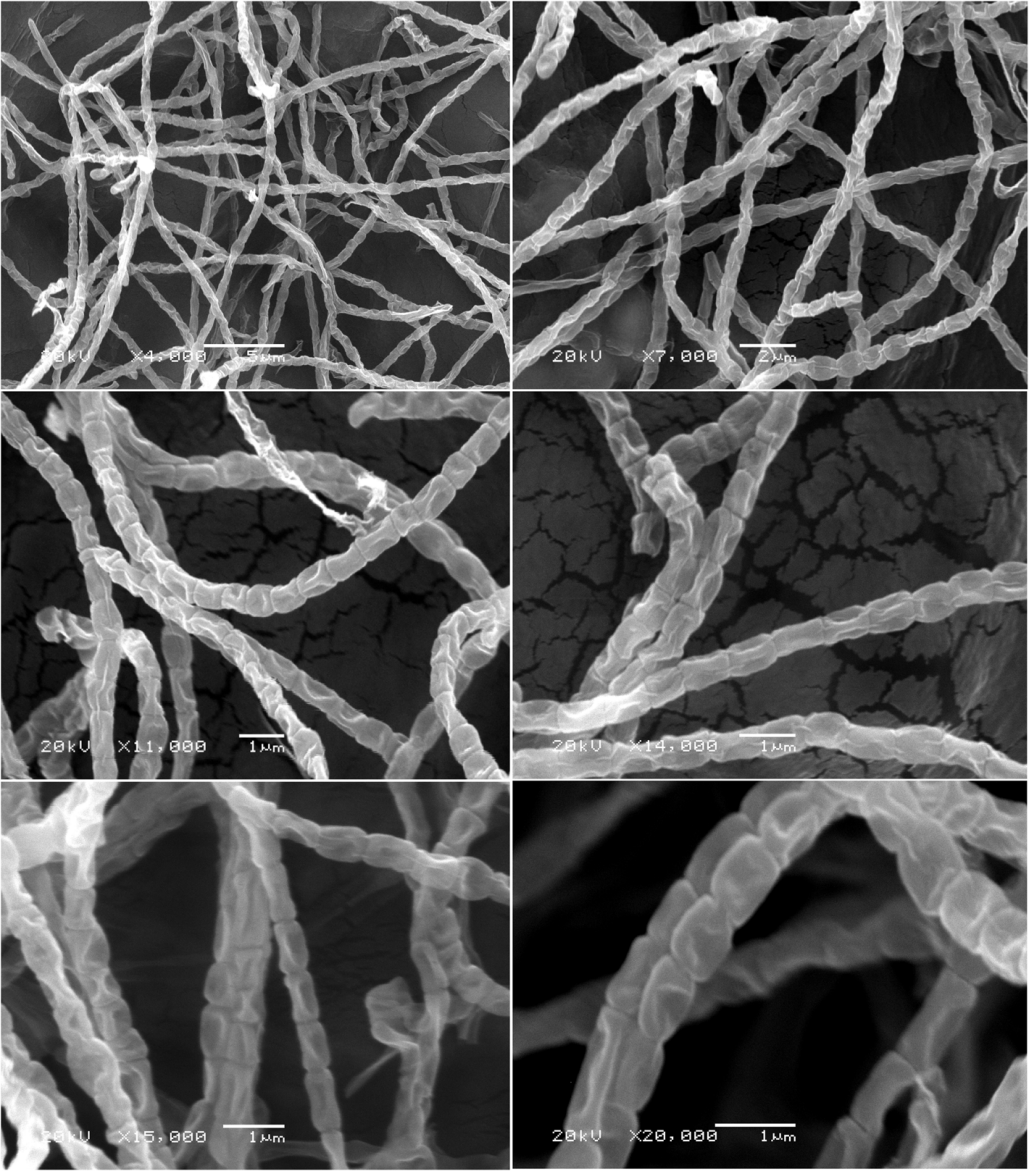
Spore-chain and spore-surface morphology of *Streptomyces* sp. strain NEAE-94 under scanning electron microscope at magnification of 4000X-20,000X

Morphological characteristics of *Streptomyces* sp. strain NEAE-94 was observed by scanning electron micrograph after incubation on medium of starch nitrate agar medium at 30°C for 14 days. Microscopic observation of *Streptomyces* sp. strain NEAE-94 showed rectiflexibiles spores chains (Fig. 3). In general, chains of mature spores are long. Spore shape is elongated (0.593–0.754 × 0.995–1.341 μm), irregular and the spore surface is smooth (Fig. 3).

### 16S rRNA gene sequence analysis and phylogenetic analysis

**Fig. 4:**
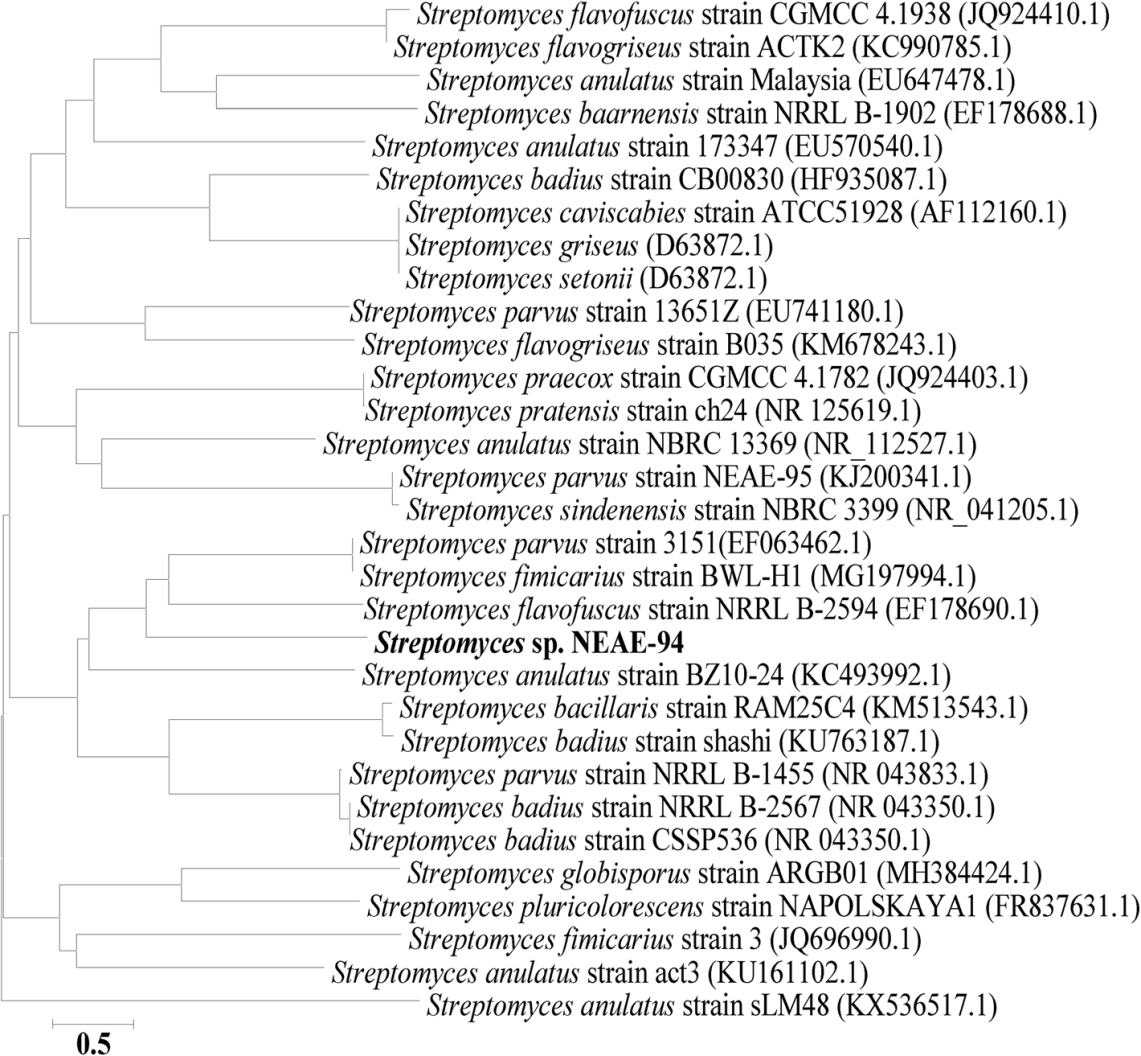
Phylogenetic tree obtained by Neighbour-joining method showing the relationship between *Streptomyces* sp. strain NEAE-94 and other related species of *Streptomyces* based on the 16S rRNA sequences

The obtained 16S rRNA sequence of *Streptomyces* sp. strain NEAE-94 was determined which gave an almost complete sequence with 1536 bp and further subjected to the BLAST search of the GenBank database and the results showed homologies with other relevant sequences of many species belonging to the *Streptomyces* genus. The phylogenetic tree (Fig. 4) showed that *Streptomyces* sp. strain NEAE-94 shared gene similarity of 99.38% to that of *Streptomyces anulatus* strain BZ10–24, query cover 94% (GenBank accession no. KC493992.1); 99.59% to that of *Streptomyces parvus* strain 3151, query cover 94% (GenBank accession no. EF063462.1); 99.38% to that of *Streptomyces flavofuscus* strain NRRL B-2594, query cover 94% (GenBank accession no. EF178690.1) and 99.19% to that of *Streptomyces fimicarius* strain BWL-H1, query cover 95% (GenBank accession no. MG197994.1).

The whole morphological and physiological properties of *Streptomyces* sp. strain NEAE-94 and its closest phylogenetic neighbors of the genus *Streptomyces* which showed significant similarities are shown in Table 1. The strain (*Streptomyces anulatus*) has been deposited in the Culture Collection Ain Shams University (CCASU). The culture collection accession number CCASU 20202 is assigned to the deposited strain.

### Screening of significant factors for production of cholesterol oxidase using Plackett–Burman design

The design matrix used for screening of the significant factors for production of cholesterol oxidase and the appropriate responses are shown in Table 2. The mycelial growth of *Streptomyces anulatus* strain NEAE-94 during cholesterol oxidase production in shake flask in submerged fermentation is shown in Fig. 2d. The results showed broad variability of the cholesterol oxidase activity (0.87 to 11.03 U/mL) reflecting the significance of the medium optimization for enhanced cholesterol oxidase production. The highest production of cholesterol oxidase (11.03 U/mL) was achieved in the run no. 18 using 50 mL medium/250 mL conical flask consists of (g/L): Glucose 5; starch 10; cholesterol 3; yeast extract 4; peptone 4; (NH_4_)_2_SO_4_ 4; FeSO_4_.7H_2_O 0.01; MgSO_4_.7H_2_O 0.5; NaCl 0.5; K_2_HPO_4_ 1 and pH 7; inoculum size was 4% (v/v) and incubated for 5 days at 37°C using an agitation speed of 150 rpm. In the run no. 4, the lowest production of cholesterol oxidase was obtained (0.87 U/mL) using 50 mL medium/250 mL conical flask consists of (g/L): Glucose 5; starch 10; cholesterol 1; yeast extract 1; peptone 1; (NH_4_)_2_SO_4_ 4; FeSO_4_.7H_2_O 0.05; MgSO_4_.7H_2_O 0.1; NaCl 1; K_2_HPO_4_ 1 and pH 9; inoculum size was 4% (v/v) and incubated for 7 days at 37°C using an agitation speed of 100 rpm.

**Table 2:** Twenty-trial Plackett–Burman experimental design used for cholesterol oxidase production

Std	Run no.	Coded levels of independent variables																			Cholesterol oxidase activity (U/mL)		Residuals
		Temperature (°C)	Incubation time (Days)	Inoculum size % (v/v)	Agitation speed (rpm)	pH	Glucose (g/L)	Starch (g/L)	Cholesterol (g/L)	Peptone (g/L)	Yeast extract (g/L)	(NH_**4**_)_**2**_SO_**4**_(g/L)	K_**2**_HPO_**4**_(g/L)	NaCl(g/L)	MgSO_**4**_.7H_**2**_O(g/L)	FeSO_**4**_.7H_**2**_O(g/L)	D_**1**_	D_**2**_	D_**3**_	D_**4**_	Actual value	Predicted value	
6	1	−1	−1	1	1	−1	1	1	−1	−1	1	1	1	1	-1	1	-1	1	-1	-1	3.39	3.71	−0.32
12	2	−1	1	− 1	1	− 1	− 1	− 1	− 1	1	1	− 1	1	1	− 1	− 1	1	1	1	1	4.60	4.78	−0.18
4	3	1	1	−1	1	1	− 1	−1	1	1	1	1	−1	1	−1	1	−1	− 1	−1	− 1	9.72	9.57	0.15
15	4	1	1	1	−1	1	−1	1	−1	− 1	− 1	−1	1	1	−1	1	1	−1	−1	1	0.87	0.72	0.15
20	5	−1	−1	− 1	− 1	−1	− 1	−1	− 1	− 1	− 1	− 1	− 1	− 1	− 1	−1	− 1	−1	− 1	−1	3.15	3.12	0.03
7	6	−1	−1	− 1	1	1	−1	1	1	−1	−1	1	1	1	1	−1	1	−1	1	−1	10.11	9.64	0.47
2	7	−1	1	1	− 1	− 1	1	1	1	1	−1	1	−1	1	−1	−1	− 1	−1	1	1	2.82	2.43	0.39
18	8	−1	−1	1	1	1	1	−1	1	−1	1	−1	− 1	−1	− 1	1	1	−1	1	1	9.67	9.40	0.27
16	9	1	1	1	1	−1	1	−1	1	− 1	− 1	− 1	− 1	1	1	−1	1	1	− 1	−1	4.12	4.52	−0.39
10	10	−1	1	−1	− 1	−1	− 1	1	1	−1	1	1	−1	−1	1	1	1	1	−1	1	6.74	7.02	− 0.28
14	11	1	1	−1	1	−1	1	−1	− 1	− 1	−1	1	1	−1	1	1	−1	−1	1	1	2.11	1.85	0.26
1	12	1	1	−1	−1	1	1	1	1	−1	1	−1	1	−1	−1	− 1	−1	1	1	−1	7.35	7.53	−0.19
17	13	−1	1	1	1	1	−1	1	−1	1	−1	−1	− 1	−1	1	1	−1	1	1	−1	2.57	2.71	−0.14
8	14	−1	−1	−1	− 1	1	1	−1	1	1	−1	−1	1	1	1	1	−1	1	−1	1	5.21	5.68	−0.47
11	15	1	−1	1	−1	− 1	−1	− 1	1	1	−1	1	1	−1	−1	1	1	1	1	−1	6.78	6.84	−0.07
5	16	−1	1	1	−1	1	1	−1	−1	1	1	1	1	−1	1	−1	1	−1	− 1	−1	1.97	1.74	0.23
19	17	1	−1	−1	1	1	1	1	−1	1	−1	1	−1	− 1	−1	− 1	1	1	−1	1	4.49	4.72	−0.23
3	18	1	−1	1	1	−1	− 1	1	1	1	1	−1	1	−1	1	−1	−1	− 1	−1	1	11.03	10.91	0.12
13	19	1	−1	1	−1	1	−1	−1	− 1	−1	1	1	−1	1	1	−1	−1	1	1	1	3.95	4.19	−0.24
9	20	1	−1	−1	−1	− 1	1	1	−1	1	1	−1	−1	1	1	1	1	−1	1	−1	2.77	2.32	0.45
**Variable level**		**A**	**B**	**C**	**D**	**E**	**F**	**G**	**H**	**J**	**K**	**L**	**M**	**N**	**O**	**P**							
−1		30	5	2	100	7	5	5	1	1	1	4	0.5	0.5	0.1	0.01							
1		37	7	4	150	9	10	10	3	4	4	8	1.0	1.0	0.5	0.05							

“Std” is the standard order and “D” means dummy

**Fig. 5:**
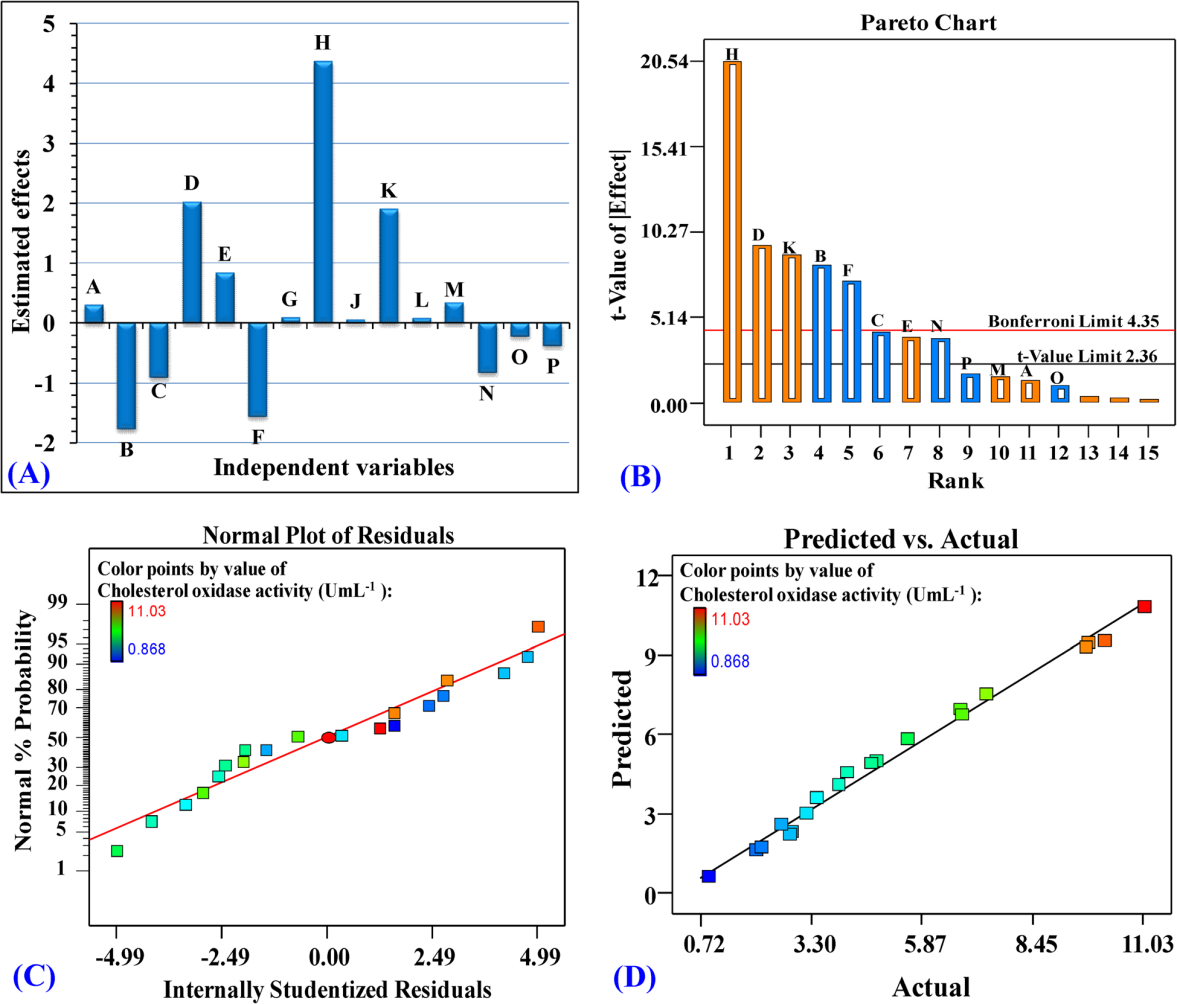
**a** The main effects of the variables, **b** The Pareto chart shows the order of significance of each variable, **c** The normal probability plot of the residuals, **d** Correlation between the experimented and predicted values for cholesterol oxidase production by *Streptomyces anulatus* strain NEAE-94 determined by the first-order polynomial equation

Table 3 shows the statistical analysis of the results of the Plackett–Burman design. Figure 5a shows the main effect of the individual independent factors on the cholesterol oxidase production. Figure 5a revealed that, temperature, agitation speed, pH, starch, cholesterol, peptone, yeast extract, ammonium sulphate and K_2_HPO_4_ positively affect cholesterol oxidase production, whereas the remaining factors named incubation time, glucose, inoculum size, MgSO_4_, NaCl and FeSO_4_ negatively affect cholesterol oxidase production. The data revealed that, starch (G), peptone (K) and ammonium sulphate (L) with higher *P*-values (0.9271, 0.9573, 0.9370; respectively), lower effects (0.09, 0.05 and 0.07; respectively) and lower contribution % (0.04, 0.02 and 0.04; respectively) are insignificant factors. The Pareto chart shows absolute effects values and illustrates the significance order of the factors that influence cholesterol oxidase production. The Pareto chart shows a reference line, any absolute effect value extending past this reference line is highly essential (Fig. 5b). Figure 5c displays a normal probability plot of the residuals. Figure 5d shows the plot of the predicted cholesterol oxidase production versus actual values.

**Table 3:** Statistical analysis of Plackett-Burman design

Term	Coefficient	Effect	% Contribution	***F-***value	***P***-value Prob > ***F***
Intercept	5.17			573.21	0.0001*
A-Temperature	0.15	0.30	0.25	17.15	0.0256*
B-Incubation time	−0.88	−1.77	8.86	615.20	0.0001*
C-Inoculum size	−0.45	−0.91	2.33	161.94	0.0010*
D-Agitation speed	1.01	2.02	11.58	804.04	< 0.0001*
E-pH	0.42	0.84	2.00	138.84	0.0013*
F-Glucose	−0.78	−1.56	6.92	480.30	0.0002*
G-Starch	0.04	0.09	0.02	9.87 × 10^−3^	0.9271*
H-Cholesterol	2.18	4.37	54.13	3757.27	< 0.0001*
J-Peptone	0.02	0.05	0.01	3.38 × 10^−3^	0.9573
K-Yeast extract	0.95	1.90	10.20	707.66	0.0001*
L-(NH_4_)_2_SO_4_	0.04	0.07	0.02	7.37 × 10^− 3^	0.9370
M- K_2_HPO_4_	0.17	0.34	0.33	22.89	0.0174*
N-NaCl	−0.41	−0.83	1.95	135.29	0.0014*
O- MgSO_4_.7H_2_O	−0.11	− 0.23	0.14	9.98	0.0509
P- FeSO_4_.7H_2_O	−0.19	−0.38	0.40	27.95	0.0132*
PRESS	12.92		Adeq Precision		79.34
C.V. %	3.08		Pred R^2^		0.9261
Mean	5.17		Adj R^2^		0.9978
Std. Dev.	0.16		R^2^		0.9996

* Significant values, “*F*: Fishers’s function, PRESS is the predicted residual sum of squares, C.V. % is the coefficient of variation%”

The values of the determination coefficient (R^2^ = 0.9996) and the adjusted determination coefficient (Adj. R^2^ = 0.9978) are very high. Cholesterol, agitation speed with a *P*-value of < 0.0001 was determined to be the most significant factors, followed by the concentration of yeast extract, incubation time (0.0001) then glucose (0.0002) (Table 3). The *P*-value < 0.05 (0.0001) implies that the model terms are significant. The *F-*value of the model (573.21) means that it is significant. The first order polynomial equation representing the production of cholesterol oxidase in relation to the independent factors was obtained by neglecting the insignificant factors:

1 $$ \mathrm{Y}=5.17+0.15\ \mathrm{A}-0.88\ \mathrm{B}-0.45\ \mathrm{C}+1.01\ \mathrm{D}+0.42\ \mathrm{E}-0.78\ \mathrm{F}+2.18\ \mathrm{H}+0.95\ \mathrm{K}+0.17\ \mathrm{M}-0.41\ \mathrm{N}-0.11\ \mathrm{O}-0.19\ \mathrm{P} $$

Where Y is the production of cholesterol oxidase and A,B,C,D,E,F,H,K,M,N,O,P are temperature, the time of incubation, size of inoculum, speed of agitation, pH, concentration of glucose, concentration of cholesterol, concentration of yeast extract, concentration of K_2_HPO_4_, NaCl, MgSO_4_ and FeSO_4_; respectively.

For assessment of Plackett-Burman design precision, the following production medium was used for cholesterol oxidase production (g/L): FeSO_4_.7H_2_O 0.01; MgSO_4_.7H_2_O 0.5; NaCl 0.5; K_2_HPO_4_ 1; yeast extract 4; cholesterol 3 and pH 7. The production medium was inoculated with inoculum size of 4% (v/v) and incubated at a temperature of 37°C in a shaker incubator at 150 rpm for 5 days. The production of cholesterol oxidase using the previous medium was 11.03 U/mL, which was increased 2.45 times compared to the enzyme activity obtained before application of Plackett-Burman design (4.51 U/mL).

### Optimization of the selected significant variables by Box–Behnken design

Box–Behnken design was used to obtain the optimal levels of the most significant factors influencing cholesterol oxidase production by *Streptomyces anulatus* strain NEAE-94 and to study the interaction effects among these factors. In the current study, 15 experiments with various combinations of agitation speed, cholesterol concentration and yeast extract concentration were performed and the experimental and predicted cholesterol oxidase production and residuals for the 15 trials are provided in Table 4.

**Table 4:** Three-level Box-Behnken design representing cholesterol oxidase production as influenced by agitation speed (X_1_), cholesterol concentration (X_2_) and yeast extract concentration (X_3_)

Std	Run	Agitation speed		Cholesterol concentration		Yeast extract concentration		Cholesterol oxidase activity (U/mL)		Residuals
		Coded	Actual (rpm)	Coded	Actual (g/L)	Coded	Actual (g/L)	Experimental	Predicted	
15	1	0	150	0	4	0	5	26.44	26.55	−0.10
13	2	0	150	0	4	0	5	25.88	26.55	−0.66
1	3	−1	100	−1	2	0	5	12.90	12.97	−0.07
9	4	0	150	−1	2	−1	3	10.31	10.50	−0.19
2	5	1	200	−1	2	0	5	11.48	10.74	0.74
7	6	−1	100	0	4	1	7	13.13	12.58	0.55
3	7	−1	100	1	6	0	5	16.29	17.03	−0.74
12	8	0	150	1	6	1	7	17.76	17.57	0.19
11	9	0	150	−1	2	1	7	8.44	8.91	−0.47
10	10	0	150	1	6	−1	3	15.03	14.56	0.47
4	11	1	200	1	6	0	5	19.47	19.40	0.07
14	12	0	150	0	4	0	5	27.31	26.55	0.77
5	13	−1	100	0	4	−1	3	5.64	5.38	0.27
6	14	1	200	0	4	−1	3	11.40	11.95	−0.55
8	15	1	200	0	4	1	7	5.89	6.16	−0.27

“Std” is the standard order

Based on the variations in the agitation speed, cholesterol concentration and yeast extract concentration, the results showed variations in the cholesterol oxidase production. Cholesterol oxidase production ranged from 5.64–27.31 U/mL. The lowest cholesterol oxidase production by *Streptomyces anulatus* strain NEAE-94 (5.64 U/mL) was achieved in the 13th run when the agitation speed was 100 rpm, cholesterol concentration was 4 g/L and yeast extract concentration was 3 g/L. The maximum value of cholesterol oxidase production was achieved in the 12th run with value of 27.31 U/mL, when agitation speed was 150 rpm, cholesterol concentration was 4 g/L and yeast extract concentration was 5 g/L.

### The analysis of variance (ANOVA) for multiple regression analysis

Table 5 contains multiple regression analysis and ANOVA for the results of the Box–Behnken design. The ANOVA of the multiple regression analysis show the model to be highly significant, as can be seen from the low probability value (< 0.0001) and the value of Fisher’s *F*-test (113.82) (Table 5). The current R^2^ and adjusted R^2^ values are 0.9951 and 0.9864; respectively. While, predicted R^2^ value is 0.9427. Accuracy and reliability of the model can be seen in the small percentage of the coefficient of variation value (CV = 5.44%), mean value (15.16), adequate precision value (31.469), PRESS value (40.03) and standard deviation value (0.82) (Table 5).

**Table 5:** Regression statistics and analysis of variance (ANOVA) for Box-Behnken design results

Source	***df***	Coefficient estimate	***F-***value	***P-***value ***Prob*** > ***F***
Model	9	26.55	113.82	< 0.0001*
X_1_	1	0.04	0.02	0.9071
X_2_	1	3.18	119.05	0.0001*
X_3_	1	0.35	1.48	0.2782
X_1_ X_2_	1	1.15	7.80	0.0383*
X_1_ X_3_	1	−3.25	62.24	0.0005*
X_2_ X_3_	1	1.15	7.80	0.0383*
X_1_^2^	1	−7.69	321.59	< 0.0001*
X_2_^2^	1	−3.82	79.45	0.0003*
X_3_^2^	1	−9.84	526.73	< 0.0001*
R^2^	0.9951	Std. Dev.	0.82	
Adj R^2^	0.9864	Mean	15.16	
Pred R^2^	0.9427	C.V. %	5.44	
Adeq Precision	31.469	PRESS	40.03	

* Significant values, “*df*: Degree of freedom, *F*: Fishers’s function, *P*: Level of significance, C.V: Coefficient of variation”

The significance of each coefficient was defined in terms of both *P* and *F* values listed in Table 5. It can be seen from the *P*-values and *F*-values that the linear coefficients of cholesterol concentration, interaction between the agitation speed and cholesterol concentration; agitation speed and yeast extract concentration; cholesterol concentration and yeast extract concentration and quadratic effects of agitation speed, cholesterol concentration and yeast extract concentration are significant as it is evident from the *F*-values of 119.05, 7.80, 62.24, 7.80, 321.59, 79.45, 526.73; respectively, and *P*-values of 0.0001, 0.0383, 0.0005, 0.0383, < 0.0001, 0.0003, < 0.0001; respectively. On the other hand, *P*-values of the linear coefficients of agitation speed (X_1_) and yeast extract concentration (X_3_) indicate that they had nonsignificant effects on cholesterol oxidase production by the strain under study.

The quadratic model of Box–Behnken design used for cholesterol oxidase production by *Streptomyces anulatus* strain NEAE-94, with a non-significant lack of fit (*F*-value 1.51 and *P*-value = 0.4219) and a very low *P*-value< 0.0001 was shown in the fit summary results (Supplementary Table 2). The largest adjusted and predicted R^2^ of 0.9864 and 0.9427 and the lowest standard deviation (0.82) was reported in the summary statistics of the quadratic model.

The optimum levels of agitation speed, cholesterol concentration and yeast extract concentration giving the maximum cholesterol oxidase production was evaluated by a second-order polynomial equation. Cholesterol oxidase production can be predicted by applying the following second-order regression equation in terms of the independent variables:

2 $$ \mathrm{Y}=26.55+0.04{\mathrm{X}}_1+3.18{\mathrm{X}}_2+0.35{\mathrm{X}}_3+1.15{\mathrm{X}}_1\ {\mathrm{X}}_2-3.25{\mathrm{X}}_1{\mathrm{X}}_3+1.15{\mathrm{X}}_2{\mathrm{X}}_3-7.69{{\mathrm{X}}_1}^2-3.82{{\mathrm{X}}_2}^2-9.84{{\mathrm{X}}_3}^2 $$

Where Y is the cholesterol oxidase production, X_1_ is the coded value of agitation speed, X_2_ is the coded value of cholesterol concentration and X_3_ is the coded value of yeast extract concentration.

### Three dimensional (3D) surface and contour plots

**Fig. 6:**
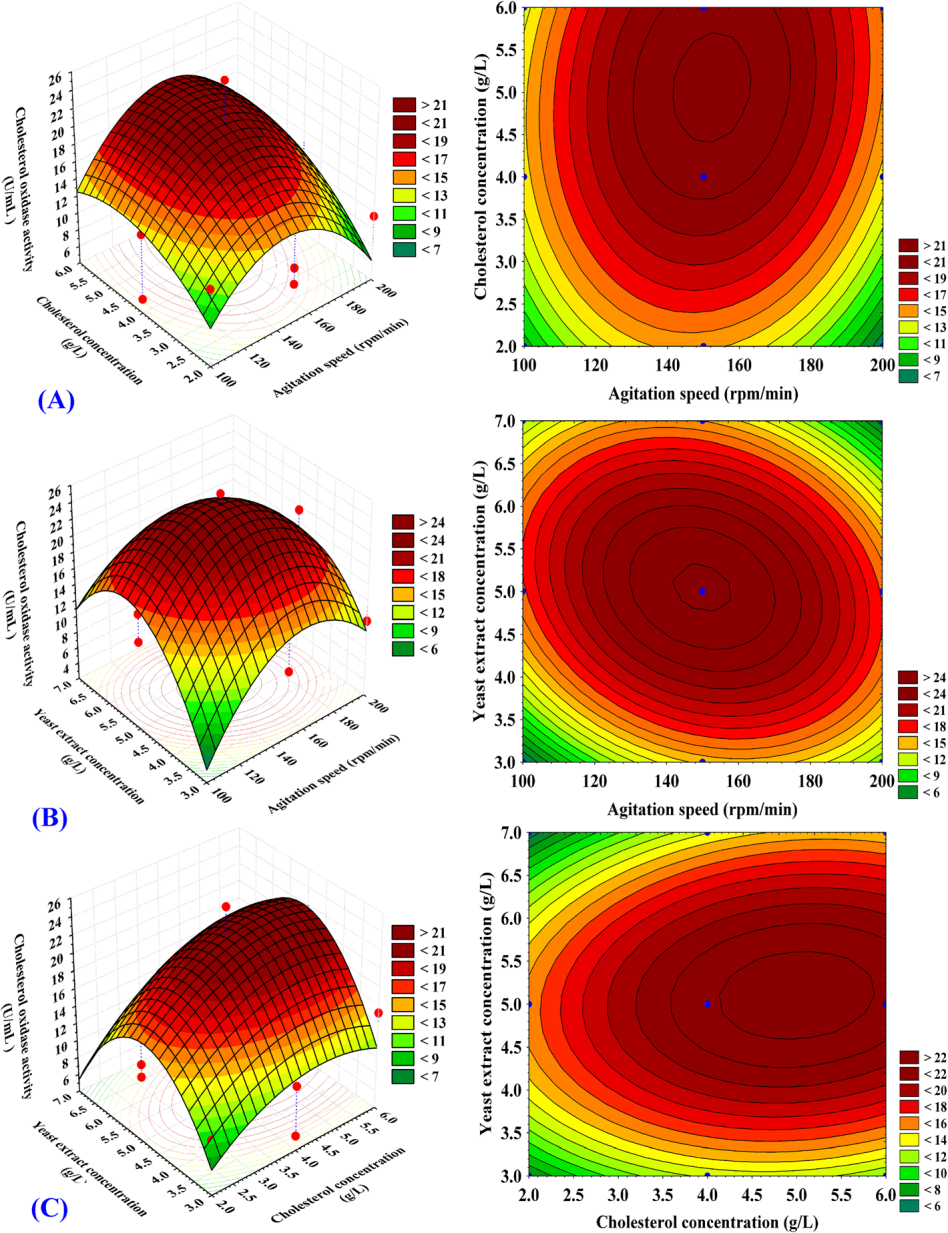
**a-c** Three-dimensional response surface plots showing the effect of agitation speed, cholesterol, yeast extract concentration and their mutual effects on the cholesterol oxidase production

To understand the interaction among the three factors (X_1_ - X_3_) and the optimum level of each factor required for the maximum cholesterol oxidase production, the 3D curves and its corresponding contour plots were generated by plotting the cholesterol oxidase production on the Z axis versus two factors are allowed to vary and the third variable is fixed at its zero level (shown in Fig. 6a–c). Figure 6a represents the cholesterol oxidase production as the simultaneous effect of agitation speed (X_1_), cholesterol concentration (X_2_) while yeast extract was kept at the central point (5 g/L). The cholesterol oxidase activity increases gradually by increasing cholesterol concentration and agitation speed till reach its optimum, but further increase in both cholesterol concentration and agitation speed leads to decrease in cholesterol oxidase activity. By solving the Eq. (2), the highest cholesterol oxidase production of 27.21 U/mL could be reached using 5 g/L yeast extract at the optimal predicted levels of agitation speed and cholesterol concentration of 150 rpm and 4.8 g/L; respectively.

The three-dimensional surface and contour plots in Fig. 6b illustrates cholesterol oxidase production as a function of agitation speed (X_1_) and yeast extract concentration (X_3_) while cholesterol concentration (X_2_) was fixed at the central point (4 g/L). Figure 6b indicates that low agitation speed (X_1_) results in lower cholesterol oxidase production and with increasing the agitation speed, the cholesterol oxidase production increases beyond 150 rpm after which cholesterol oxidase production was reduced. Lower and higher concentrations of yeast extract (X_3_) results in lower cholesterol oxidase production, and the maximum cholesterol oxidase production, obviously obtained at the central level of the yeast extract concentration. By analysis of Fig. 6b and solving the Eq. (2), the maximum predicted cholesterol oxidase production of 26.55 U/mL could be reached using 4 g/L cholesterol at the optimal predicted levels of agitation speed (150 rpm) and yeast extract concentration (5 g/L).

Figure 6c shows cholesterol oxidase production as influenced by cholesterol concentration (X_2_) and the concentration of yeast extract (X_3_) by maintaining the agitation speed at the central point (150 rpm). With an increased concentrations of both cholesterol and yeast extract, cholesterol oxidase production by the selected strain (*Streptomyces anulatus* strain NEAE-94) was improved and the maximum cholesterol oxidase production was obtained at the middle levels of two factors, and further increase of cholesterol concentration or yeast extract concentration decreases cholesterol oxidase activity. By analysis of Fig. 6c and solving the Eq. (2), the maximal predicted cholesterol oxidase production of 27.21 U/mL could be reached using agitation speed (150 rpm) and the optimal predicted levels of 4.8 g/L cholesterol and yeast extract concentration (5 g/L).

### Verification of the model

Using the optimal levels of the process variables as obtained using Box–Behnken design, the experimental cholesterol oxidase production was verified and compared with the predicted value of cholesterol oxidase production (26.55 U/mL). The maximum experimental cholesterol oxidase production by *Streptomyces anulatus* strain NEAE-94 was 27.31 U/mL. The verification revealed a high degree of model accuracy (97.21%).

## Discussion

### Taxonomic conclusions

*Streptomyces* sp. strain NEAE-94 mainly have the same characteristics as *Streptomyces anulatus*, *Streptomyces flavofuscus*, *Streptomyces parvus* and *Streptomyces fimicarius* in that it produced rectiflexibiles spore chains and did not produce melanin pigments. However, *Streptomyces* sp. strain NEAE-94 mainly differed from *Streptomyces fimicarius* in that it produces yellow aerial mycelium, yellow substrate mycelium, and yellow diffusible pigment on ISP medium 2. On the other hand, *Streptomyces fimicarius* lost the ability to produce a diffusible pigment and not produce distinctive substrate mycelium pigment. Also *Streptomyces* sp. strain NEAE-94 differed from *Streptomyces fimicarius* and *Streptomyces parvus* in the pattern of utilization of carbon sources (Table 1). Moreover, *Streptomyces* sp. strain NEAE-94 mainly differed from *Streptomyces flavofuscus* in the diffusible pigment and color of substrate mycelium. The phylogenetic tree (Fig. 4) was constructed by using the tree-making neighbor-joining algorithm method of Saitou and Nei [21] using MEGA 3.0 software [22]. The comparative study between the related species of the genus *Streptomyces* and *Streptomyces* sp. strain NEAE-94 (Table 1) indicated that it mostly related to *Streptomyces anulatus* [20]. Accordingly, *Streptomyces* sp. strain NEAE-94 was identified as *Streptomyces anulatus* strain NEAE-94 (accession number is KC354803).

### Screening of significant factors for production of cholesterol oxidase using Plackett–Burman design

The values of the determination coefficient (R^2^ = 0.9996) and the adjusted determination coefficient (Adj. R^2^ = 0.9978) are very high and suggests a strong model significance [23]. The smaller *P*-value of the factor reveals that the factor is more essential for cholesterol oxidase production.

The residuals’ normal probability plot is a valuable tool for detecting and explaining the systemic deviations from the normality [24]. Figure 5c displays a normal probability plot of the residuals. The residuals have been drawn against a theoretical normal model distribution in such a manner that the points for cholesterol oxidase production should form an approximately straight line. Departures from this straight line show deviations from normality. The normal probability plot of the residuals shows points close a diagonal line; so that residuals seem to be distributed nearly normal. This means the model was well designed to the findings of the experiments. Figure 5d shows the plot of the predicted cholesterol oxidase production versus actual values, while the dots collected around a diagonal line reveals the model’s excellent fit.

### Optimization of the selected significant variables by Box–Behnken design

Depending on the statistical analysis of Plackett–Burman design results, the factors with positive impact on production of cholesterol oxidase (temperature, agitation speed, pH, starch, cholesterol, peptone, yeast extract, ammonium sulphate and K_2_HPO_4_) have been maintained at their highest levels (+ 1). Whereas, the factors with negative impact on cholesterol oxidase production (incubation time, inoculum size, MgSO_4_, NaCl and FeSO_4_) were fixed at their low (− 1) levels. On the other hand, the insignificant factors (starch, peptone and (NH_4_)_2_SO_4_) have been omitted in the subsequent experiments.

The maximum cholesterol oxidase production obtained from this study (27.31 U/mL) by *Streptomyces anulatus* strain NEAE-94 is superior and greater than most of the other reported values such as the cholesterol oxidase production by *Rhodococcus equi* no. 23 (0.24 U/mL) [25], *Micrococcus* sp. (3.68 U/mL) [26], *Streptomyces* A (2.44 U/mL) [27], *Brevibacterium* sp. (1.483 U/mL) [28], *Streptomyces lavendulae* (2.21 U/mL) [29], *Streptomyces* sp. (6.2 U/mL) [30], *Bacillus cereus* (1.67 U/mL) [31] and *Streptomyces fradiae* (0·03 U/mL) [32].

The current R^2^ and adjusted R^2^ values were 0.9951 and 0.9864; respectively, implying that the model is appropriate to represent the real relationship between cholesterol oxidase production and the selected factors. The largest R^2^ value showed that experimental and expected cholesterol oxidase production values are in excellent agreement [33]. Predicted R^2^ value of 0.9427 shows that the model is sufficient to predict the value of the production of cholesterol oxidase in the range of factors used.

The non- significant lack-of-fit, the high value of adjusted and predicted R-squared, low PRESS value, high *F*-value, low standard deviation and high adequate precision indicating the validity and high degree of accuracy of the model prediction for the production of cholesterol oxidase by *Streptomyces anulatus* strain NEAE-94.

Enhanced production of cholesterol oxidase was recorded with the use of different compounds as cholesterol, yeast extract [32]; malt extract, potato starch and peptone [34] as substrates. Yehia et al. [35] noted that the growth and breakdown of cholesterol by the tested bacterial isolates were largely and severely affected by cholesterol concentration in the culture medium. The largest cholesterol breakdown by the *Enterococcus hirae* was achieved using 1 g/L cholesterol. Sojo et al. [36] and Yazdi et al. [32] reported that the largest cholesterol breakdown and maximum cholesterol oxidase production by the *Rhodococcus erythropolis* and *Streptomyces fradiae* was achieved using 2 g/L cholesterol.

There are different and opposite effects of nitrogen sources on the production of cholesterol oxidase in the literature. Voelker and Altaba [37] recorded an increase in the production of cholesterol oxidase by organic nitrogen much greater than inorganic nitrogen. The reason is that organic nitrogen may contain the most types of growth factors and amino acids required for the microbial growth and could be immediately metabolized by cells, thereby supporting the cholesterol oxidase production [29]. Maximum cholesterol oxidase production by *Rhodococcus equi* 2C and *Rhodococcus equi* no. 23 was recorded using 0.3% [32] and 0.4–0.5%, w/v yeast extract [25]; respectively. On the other hand, Moradpour et al. [38] and Ahmad [39] reported that the best sources of nitrogen for cholesterol decomposition by *Pseudonocardia compacta* S-39 were ammonium nitrate, ammonium sulphate and sodium nitrate [40]. Liu et al. [41] reported that the best sources of nitrogen for maximum cholesterol oxidase production by *Arthrobacter simplex* were ammonium salts.

Appropriate oxygen must be given in the fermentation media using shaken cultures to satisfy the organism’s growing demands and to produce the desired end product. The suitable agitation speed ensures that the dissolved oxygen in the medium is adequately supplied and can become vital for microbial biosynthesis of certain end products.

In this study, the maximum cholesterol oxidase production (27.31 U/mL) is obtained using the following medium formula (g/L): yeast extract 5, cholesterol 4, FeSO_4_.7H_2_O 0.01, MgSO_4_.7H_2_O 0.5, NaCl 0.5, K_2_HPO_4_ 1, pH 7, inoculum size 4% (v/v), temperature 37°C, agitation speed 150 rpm, medium volume 50 mL and incubation time 5 days.

El-Naggar et al. [14] used the 2-level Plackett–Burman experimental design in 20 experimental run to evaluate the importance of 15 process variables of medium components and operating conditions for the production of cholesterol oxidase by *Streptomyces cavourensis* strain NEAE-42. The most important factors that significantly influenced cholesterol oxidase production were initial pH, cholesterol and (NH_4_)_2_SO_4_ concentrations. El-Naggar et al. [14] reported that the optimal levels of the three selected process factors for maximum cholesterol oxidase production (20.521 U/mL) as obtained from the central composite design were pH 8; cholesterol concentration 3 g/L; (NH_4_)_2_SO_4_ 8 g/L. However, Ahmad and Goswami [42] optimized the medium for production of cholesterol oxidase by *Rhodococcus* sp. NCIM 2891 using the classical and statistical methods. They reported that the maximum cholesterol oxidase production (3.25 U/mL) was obtained in the statistically optimized medium under the optimal levels of the process factors that were 2.5 g/L (NH_4_)_2_HPO_4_, 9 g/L yeast extract and 3.5 g/L cholesterol in approximately 60 h of cultivation at 30°C and pH 7.0. On the other hand, Srivastava et al. [43] applied various statistical optimization techniques to enhance the cholesterol oxidase production by *Streptomyces rimosus* MTCC 10792. They reported that, out of the examined factors, yeast extract, dextrose, starch and ammonium carbonate were the most significant factors and the maximum cholesterol oxidase production was 5.41 U/mL in the optimized medium using the optimum concentrations of the four variables that were (g/100 mL medium): 0.05 ammonium carbonate, 0.1 starch, 0.8 dextrose and 0.99 yeast extract.

Moradpour et al. [38] screen various process variables that had a major impact on cholesterol oxidase production by *Streptomyces badius* using Plackett-Burman design and optimized these variables using Box-Behnken design. They reported that, yeast extract, pH, Tween 20 and temperature were the most significant factors and the maximum cholesterol oxidase production by *Streptomyces badius* was 2.05 U/mL in the optimized medium using the optimum levels of the four variables that were determined to be: yeast extract, 0.45%; pH, 6.5; Tween 20, 0.05% and 30°C. Moreover, ElBaz et al. [44] used a two-step statistical approach to optimize the production of cholesterol oxidase from *Bacillus pumilus*. The maximum cholesterol oxidase production (90 U/mL) was obtained after 6 days of fermentation at pH 8 with medium/flask ratio of 0.35 and the concentrations of cholesterol, NH_4_NO_3_, yeast extract and Tween 80 were 0.2, 0.3, 1 and 0.2%; respectively.

Kuppusamy and Kumar [31] used traditional one variable at a time method to find the key nutritional components such as different carbon sources, nitrogen sources, metal ions and different physical parameters like incubation time, temperature and pH to enhance cholesterol oxidase production by *Bacillus cereus* strain KAVK4. The highest production of cholesterol oxidase was achieved under flask conditions using the optimal levels of fructose (as a carbon source) in the production medium at a concentration of 2%, ammonium nitrate (as nitrogen source) at a concentration of 0.2% and magnesium sulphate as metal ion source at a concentration of 0.03%. The maximum production of cholesterol oxidase by *Bacillus cereus* strain KAVK4 of 1.67 U/mL was achieved at the optimum process variable values (incubation time was at 32 h, pH 7.5 at room temperature).

Yang and Zhang [28] used a three level central composite design to investigate the correlation between three independent variables (cholesterol, Tween-80 and treatment time) and cholesterol oxidase production by *Brevibacterium* sp. They found that, the optimal values of the three independent variables resulted in highest cholesterol oxidase production by *Brevibacterium* sp. (1.483 U/mL) were determined to be: 22.361 (min) treatment time, 0.2932% (v/v) Tween-80, 4.076 g/L cholesterol. In addition, El-Naggar et al. [45] used the Plackett-Burman design to evaluate the influence of nutritional and environmental variables for cholesterol oxidase production by *Streptomyces aegyptia* strain NEAE− 102. They found that, out of 15 variables screened by Plackett-Burman design experiments, pH, incubation time and cholesterol concentration were the most significant variables for cholesterol oxidase production. El-Naggar et al. [45] used a face centered central composite design to optimize the levels and analyze the combined effects of pH, incubation time and cholesterol concentration. The optimum levels of these variables for the high cholesterol oxidase production (15.631 U/mL) were determined to be: pH 6, 5 days of incubation time and 3 g/L cholesterol.

Varma and Nene [34] studied cholesterol oxidase production by *Streptomyces lavendulae* NCIM 2421. A peak of cholesterol oxidase activity of 1.8 U/mL was detected at 72 h. They found that, *Streptomyces lavendulae* NCIM 2421 is a constitutive producer of cholesterol oxidase where the addition of cholesterol to the medium did not enhance cholesterol oxidase activity. Whereas, Chauhan et al. [29] used orthogonal array method and response surface methodology to optimize medium for cholesterol oxidase production by *Streptomyces lavendulae* NCIM 2499. They reported that the model predicted maximum cholesterol oxidase production (2.21 U/mL) could be achieved after 72 h of incubation using the medium of the following composition (g/L): sodium chloride 0.7, MgSO_4_ 2, K_2_HPO_4_ 0.6, soyabean meal 20, malt extract 20, and glycerol 10 mL/L. An initial pH of 7.5 supported the maximum production of cholesterol oxidase. Moreover, Srivastava et al. [43] standardized the process of cholesterol oxidase production by studying a different range of various parameters at shake flask level. They found that the maximum cholesterol oxidase production by *Streptomyces rimosus* was achieved using the optimal levels of process variables which found to be inoculum size (3%, v/v), pH (7), incubation temperature (30°C), incubation time (48 h) and agitation speed (200 rpm). Fazaeli et al. [46] achieved maximum cholesterol oxidase production by *Escherichia coli* when the induced culture production medium was incubated for 24 h at 15°C. Also, Niwas et al. [30] studied the effect of process variables on cholesterol oxidase production by *Streptomyces* sp. at shake flask level. The results indicated that the maximum cholesterol oxidase production (6.2 U/mL) was achieved using 0.05%, w/v cholesterol, pH 7 and 35°C, 200 rpm.

## Conclusion

*Streptomyces anulatus* strain NEAE-94 could be used as a promising, efficient source for cholesterol oxidase production. The maximum cholesterol oxidase production after statistical optimization of fermentation process variables was 27.31 U/mL with a fold of increase 6.06 compared with the cholesterol oxidase production before applying Plackett-Burman design (4.51 U/mL).

## Methods

### Microorganisms and cultural conditions

*Streptomyces* sp. strain NEAE-94 was isolated from a soil sample collected from Baltim, Kafr el-Sheikh Governorate, in the north of Egypt. *Streptomyces* sp. strain NEAE-94 isolation has been made on the plates of starch-nitrate agar medium of the following constituents (g/L): FeSO_4_.7H_2_O 0.01; CaCO_3_ 3; NaCl 0.5; MgSO_4_.7H_2_O 0.5; K_2_HPO_4_ 1; KNO_3_ 2; starch 20 and agar 20 in 1 L of the distilled water. The plates were incubated for 7 days at 30°C. For long time preservation, the isolate was stored as spore suspensions in 20% (v/v) glycerol. *Streptomyces anulatus* NEAE-94 is an antagonistic actinomycete exhibited a broad antimicrobial spectrum against several bacterial strains including multidrug-resistant *Staphylococcus aureus, E. coli, Bacillus subtilis* and *Pseudomonas aeruginosa* [19].

### *Streptomyces* sp. strain NEAE-94 efficiency for cholesterol oxidase production using colony staining method

The efficiency of *Streptomyces* sp. strain NEAE-94 for the production of cholesterol oxidase was investigated on agar plate medium containing cholesterol as the sole source of carbon according to the method of El-Naggar et al. [14]. The medium contains g/L: Cholesterol 2, potassium nitrate 2, potassium phosphate dibasic 1, magnesium sulfate heptahydrate 0.5, sodium chloride 0.5, calcium carbonate 3, ferrous sulfate heptahydrate 0.01, agar 20 and distilled water 1 L; pH was adjusted to 7–7.2. A colony staining procedure was applied on the growing colonies to verify cholesterol oxidase production potential. Discs of filter papers were immersed in 100 mM potassium buffer phosphate (pH 7.0) containing 0.5% cholesterol; 6% phenol; 1.7% 4-aminoantipyrine and 3 U/mL horseradish peroxidase. After that, the soaked discs were then placed on the grown colonies and incubated again for 24 h at room temperature. Cholesterol oxidase activity was confirmed by red color development due to the formation of quinoneimine dye [47].

### Inoculum preparation and cholesterol oxidase production

Two hundred fifty millilitre Erlenmeyer flasks containing 50 mL of broth medium, comprised of g/L: (NH_4_)_2_SO_4_ 7.5; cholesterol 2; K_2_HPO_4_ 1; MgSO_4_.7H_2_O 0.5; FeSO_4_.7H_2_O 0.02; ZnSO_4_ 0.002; CaSO_4_ 0.002; MnSO_4_ 0.008; NaCl 1; CaCl_2_ 0.0002; peptone 4; yeast extract 6; starch 9; glucose 12; Tween 80 0.05) [32] were inoculated with five disks (9 mm diameter) taken from the stock culture of *Streptomyces* sp. strain NEAE-94 grown on starch nitrate agar medium. The inoculated flasks were incubated at 30°C for 48 h in rotating shaker incubator at 200 rpm, and used as inoculum for subsequent experiments.

Fifty millilitre of the fermentation medium was inoculated with the prepared inoculum and incubated in rotating shaker incubator at 30–37°C and 150–200 rpm. After the incubation time, the cell free- culture supernatant containing the crude enzyme was obtained by centrifugation of the mycelium culture using refrigerated centrifuge at 4°C and 6000×*g* for 15 min.

### Assessment of cholesterol oxidase activity

The cholesterol oxidase activity was determined spectrophotometry by the method of El-Naggar et al. [45]. One enzyme activity unit (U): is the quantity of the enzyme needed for forming one μmol H_2_O_2_ /min at 37°C.

### Cultural and morphological properties of *Streptomyces* sp. strain NEAE-94

Spore mass color, substrate mycelium color (reverse side of the colony) and soluble diffusible pigments were investigated on ISP media as described by the method of Shirling and Gottlieb [48] method. The spore chain morphology was examined on starch-nitrate agar medium with Scanning Electron Microscopy “Jeol JSM-6360 LA operates at 20 Kv”.

### Physiological properties

The ability of the strain to utilize carbon sources was determined according to the method described by Shirling and Gottlieb [48] using 13 sterilized carbon sources, namely: sucrose, ribose, D (+) glucose, L-arabinose, D (+) xylose, D (+) galactose, D (−) fructose, D (+) mannose, maltose, raffinose, rhamnose, trehalose and cellulose. Decomposition of cellulose was determined using Hutchinson liquid medium [49], cellulose was used in the final concentration of 1%, W/V, and the medium was inoculated with the spore suspension of the strain. The strain ability to reduce nitrate to nitrite was determined using Giltay liquid medium [50]. The degradation of lecithin “lecithinase activity” was determined on plates of egg–yolk medium using Nitsch and Kützner [51] method. Starch hydrolysis (amylase activity) by the strain was determined using starch-nitrate agar plates. Spore suspension was inoculated by streak the organism on the plates and incubated for 7 days at 30°C. After that, the plates were flooded with the solution of iodine. The presence of clear zone around the growth indicated positive amylase activity [52]. Test tubes containing skimmed milk were sterilized, inoculated with spore suspension and the degree of coagulation and peptonization of milk were recorded after 7 days of incubation at 30°C [53]. Liquefaction of gelatin as a proteolytic character was determined following the method described by Preobrazhenskaya [54]. Production of melanin by *Streptomyces* sp. strain NEAE-94 was determined on slants of ISP media 1, 6 and 7; the production of brown or black soluble pigments was recorded after 7 days of incubation at 30°C that indicates positive production of melanin [48]. Degradation of casein [55], L-asparaginase production [56], sodium chloride tolerance [57], chitosanase [58] and uricase activities [59] were tested. The production of antimicrobial agents by *Streptomyces* sp. strain NEAE-94 was investigated on agar plate medium according to the method of El-Naggar et al. [19].

### Molecular characterization

According to the method described by Sambrook et al. [60], the total genomic DNA of the strain was prepared. The PCR reaction was carried out using El-Naggar et al. [61] method. The amplification of 16S rRNA gene from the *Streptomyces* sp. under investigation was carried out via polymerase chain reaction (PCR) using universal primer designed to amplify the full length of the gene. The reverse primer was 1492r (5′-TACGGYTACCTTGTTACGACTT-3′) and the forward primer was 27f (5′-AGAGTTTGATCMTGCCTCAG-3′). The PCR reaction was conducted in a total volume of 100 μL that contains 0.5 μL Taq polymerase, 10 μL deoxyribonucleotide 5′-triphosphate (dNTP’s) (250 mM), 4 μL of both forward and reverse primers of 10 pmol, 1 μL template DNA 50 ng, 3.5 μL MgCl_2_ (25 Mm), 10 μL PCR buffer, up to 100 μL with water.

The amplified DNA was sequenced and the resulting sequence of 16S rRNA was deposited under accession number KC354803 in the GenBank NCBI database. 16S rRNA gene sequence (1536 bp) of *Streptomyces* sp. strain NEAE-94 was aligned with the 16S rRNA sequences of the related species of *Streptomyces* by using BLAST program [62]. The phylogenetic tree was built using version 2.1of MEGA4 software [22] via the neighbor-joining algorithm method.

### Selection of significant variables for cholesterol oxidase production by Plackett–Burman design

The Plackett-Burman statistical design [63] is an efficient method used to screen and to identify the significant variables among large number of variables that have significant effects on a process [64, 65]. Plackett-Burman design was used in this study to select the medium components and environmental conditions that had a significant effect, either negatively or positively on cholesterol oxidase production out of 15 independent variables. The Plackett-Burman experiment was conducted in 20 runs to study the effect of the selected 15 variables on cholesterol oxidase production based on their main effects. Each variable was examined in low (−) and high (+) levels. The chosen factors were glucose (5 and 10 g/L), starch (5 and 10 g/L), cholesterol (1 and 3 g/L), yeast extract (1 and 4 g/L), peptone (1 and 4 g/L), (NH_4_)_2_SO_4_ (4 and 8 g/L), FeSO_4_.7H_2_O (0.01 and 0.05 g/L), MgSO_4_.7H_2_O (0.1 and 0.5 g/L), NaCl (0.5 and 1 g/L), K_2_HPO_4_ (0.5 and 1 g/L), pH (7 and 9), inoculum size (2 and 4%, v/v), incubation time (5 and 7 days), temperature (30 and 37°C) and agitation speed (100 and 150 rpm). As well, 4 dummy variables (D_1_-D_4_) were included.

Plackett–Burman design is based on the first order model:

3 $$ Y={\beta}_0+\sum {\beta}_i{X}_i $$

Where, Y is cholesterol oxidase production, β_0_ is the model intercept, X_i_ is the level of each independent variable and *β*_*i*_ is the linear coefficient.

### Optimization of cholesterol oxidase production by Box-Behnken design (BBD)

Based on the results of Plackett-Burman experiments, three factors with the highest *P*-values, *F*-values, effects and contribution % were selected for further optimization using Box–Behnken design [66]. These factors were agitation speed (X_1_, 100–200 rpm), cholesterol concentration (X_2_, 2–6 g/L) and yeast extract concentration (X_3_, 3–7 g/L). Each factor varies on three different levels (− 1, 0, + 1), with three center points resulting in a total of 15 different experiments. BBD was used to estimate the optimal levels of these variables and to study the individual and interaction effects between the selected process variables affecting cholesterol oxidase production. Linear, quadratic and interaction effects of the three significant independent variables on cholesterol oxidase production were calculated to correlate the relationship between production of cholesterol oxidase (Y) and these factors and to predict their optimal levels using the following second order polynomial equation:

4 $$ Y={\beta}_0+\sum \limits_i{\beta}_i{X}_i+\sum \limits_{ii}{\beta}_{ii}{X_i}^2+\sum \limits_{ij}{\beta}_{ij}{X}_i{X}_j $$

In which “Y is the cholesterol oxidase production, X_i_ is the coded levels of independent variables, β_0_ is the regression coefficients, β_ij_ is the interaction coefficients, β_i_ is the linear coefficient and β_ii_ is the quadratic coefficients”.

### Statistical analysis

“The experimental designs and statistical analysis were performed using Design Expert software version 7 for Windows. The 3D surface plots were drawn using STATISTICA software version 8.”

## Supplementary information

**Additional file 1.**

## Data Availability

All data generated or analyzed during this study are included in this published article [and its supplementary information files].

## Abbreviations

rRNA: Ribosomal ribonucleic acid
FAD: Flavin adenine dinucleotide
HIV: Human immunodeficiency virus
RSM: Response surface methodology
*P*: Level of significance
R^2^: Determination coefficient
Adj. R^2^: Adjusted determination coefficient
*F*: Fisher’s function
ANOVA: Analysis of variance
CV: Coefficient of variation
PRESS: Predicted residual sum of squares
DNA: Deoxyribonucleic acid
PCR: Polymerase chain reaction
dNTP’S: Deoxyribonucleotide 5′– triphosphate
BBD: Box- Behnken design
