# Correction to: Identification of cholesterol-assimilating actinomycetes strain and application of statistical modeling approaches for improvement of cholesterol oxidase production by *Streptomyces anulatus* strain NEAE-94

**DOI:** 10.1186/s12866-020-01791-x

**Published:** 2020-04-30

**Authors:** Noura El-Ahmady El-Naggar, Nancy M. El-Shweihy

**Affiliations:** Department of Bioprocess Development, Genetic Engineering and Biotechnology Research Institute, City for Scientific Research and Technological Applications, Alexandria, Egypt

**Correction to: BMC Microbiol**

**https://doi.org/10.1186/s12866-020-01775-x**

Following publication of the original article [1], the authors reported an error in Fig. 2c. The correct Fig. 2c is presented below.

**Fig. 2:**
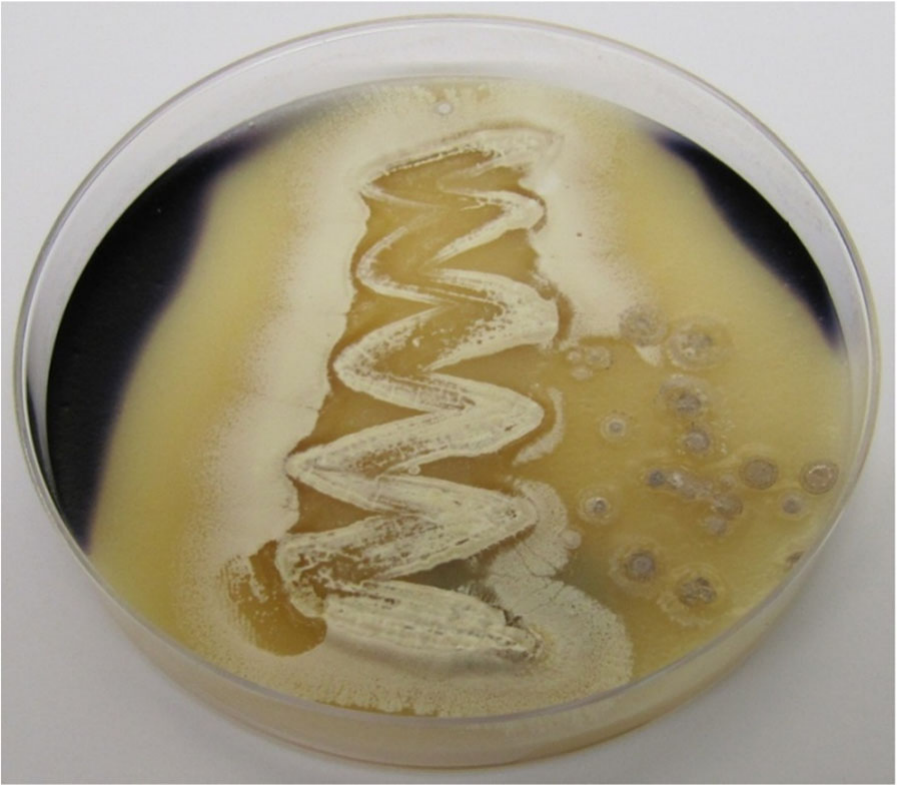
(c) plate assay showing starch hydrolysis by *Streptomyces* sp. strain NEAE-94

## References

[1] El-Naggar, El-Shweihy (2020). Identification of cholesterol-assimilating actinomycetes strain and application of statistical modeling approaches for improvement of cholesterol oxidase production by *Streptomyces anulatus* strain NEAE-94. BMC Microbiol.

