# Supplementary material for: Identification of cholesterol-assimilating actinomycetes strain and application of statistical modeling approaches for improvement of cholesterol oxidase production by Streptomyces anulatus strain NEAE-94
Source: BMC Microbiol. 2020 Apr 10;20:86. doi: 10.1186/s12866-020-01775-x (PMC7149892; doi:10.1186/s12866-020-01775-x)
Supplement: Supplementary file 1 — Additional file 1. [file 12866_2020_1775_MOESM1_ESM.docx]

**Identification of cholesterol-assimilating actinomycetes strain and application of statistical modeling approaches for improvement of cholesterol oxidase production by *Streptomyces anulatus* strain NEAE-94**

**Noura El-Ahmady El-Naggar*, Nancy M. El-Shweihy**

Department of Bioprocess Development, Genetic Engineering and Biotechnology Research Institute, City for Scientific Research and Technological Applications, Alexandria, Egypt

*To whom correspondence should be addressed.

**Dr. Noura El-Ahmady Ali El-Naggar**

**Address:**

Bioprocess Development Department,

Genetic Engineering and Biotechnology Research Institute,

City of Scientific Research and Technological Applications,

New Borg El- Arab City, 21934, Alexandria, Egypt

**Tel:** (002)01003738444

**Fax:** (002)03 4593423

**E-mail:** nouraelahmady@yahoo.com

**Supplementary Table 1.** Cultural properties of *Streptomyces* sp. strain NEAE-94

| **Medium** | **Color of** | | | **Growth** |
| --- | --- | --- | --- | --- |
|  | **Diffusible pigment** | **Substrate mycelium** | **Aerial**  **mycelium** |  |
| ISP 2 | Yellow | Yellow | Whitish yellow | Excellent |
| ISP 3 | Faint yellow | Yellow | Yellow | Excellent |
| ISP 4 | Yellow | Yellow | Yellow | Excellent |
| ISP 5 | Yellow | Faint orange | Faint yellow | Weak |
| ISP 6 | Non-pigmented | Faint orange | Faint yellow | Weak |
| ISP 7 | Yellow | Brownish orange | Yellow | Very good |

**Supplementary Table 2.** Fit summary for Box-Behnken design results

| **Sequential Model Sum of Squares** | | | | | | | | |
| --- | --- | --- | --- | --- | --- | --- | --- | --- |
| **Source** | **Sum of Squares** | ***df*** | **Mean Square** | | ***F-*value** | | ***P-*value**  ***Prob* >*F*** | |
| Linear vs Mean | 81.84 | 3 | 27.28 | | 0.49 | | 0.6986 | |
| 2FI vs Linear | 52.84 | 3 | 17.61 | | 0.25 | | 0.8594 | |
| Quadratic vs 2FI | 560.74 | 3 | 186.91 | | 275.33 | | < 0.0001* | |
| Residual | 1.04 | 2 | 0.52 | |  | |  | |
| **Lack of Fit Tests** | | | | | | | | |
| **Source** | **Sum of Squares** | ***df*** | **Mean Square** | | ***F-*value** | | ***P-*value**  ***P*rob >*F*** | |
| Linear | 615.94 | 9 | 68.44 | | 131.78 | | 0.0076* | |
| 2FI | 563.10 | 6 | 93.85 | | 180.71 | | 0.0055* | |
| Quadratic | 2.36 | 3 | 0.79 | | 1.51 | | 0.4219 | |
| Pure Error | 1.04 | 2 | 0.52 | |  | |  | |
| **Model Summary Statistics** | | | | | | | | |
| **Source** | **Standard deviation** | **R-Squared** | | **Adjusted R-Squared** | | **Predicted R-Squared** | | **PRESS** |
| Linear | 7.49 | 0.1171 | | -0.1237 | | -0.3361 | | 933.70 |
| 2FI | 8.40 | 0.1927 | | -0.4127 | | -0.9671 | | 1374.66 |
| Quadratic | 0.82 | 0.9951 | | 0.9864 | | 0.9427 | | 40.03 |
| * Significant values, “*df* : degree of freedom, PRESS: sum of squares of prediction error, two factors interaction: 2FI” | | | | | | | | |

**
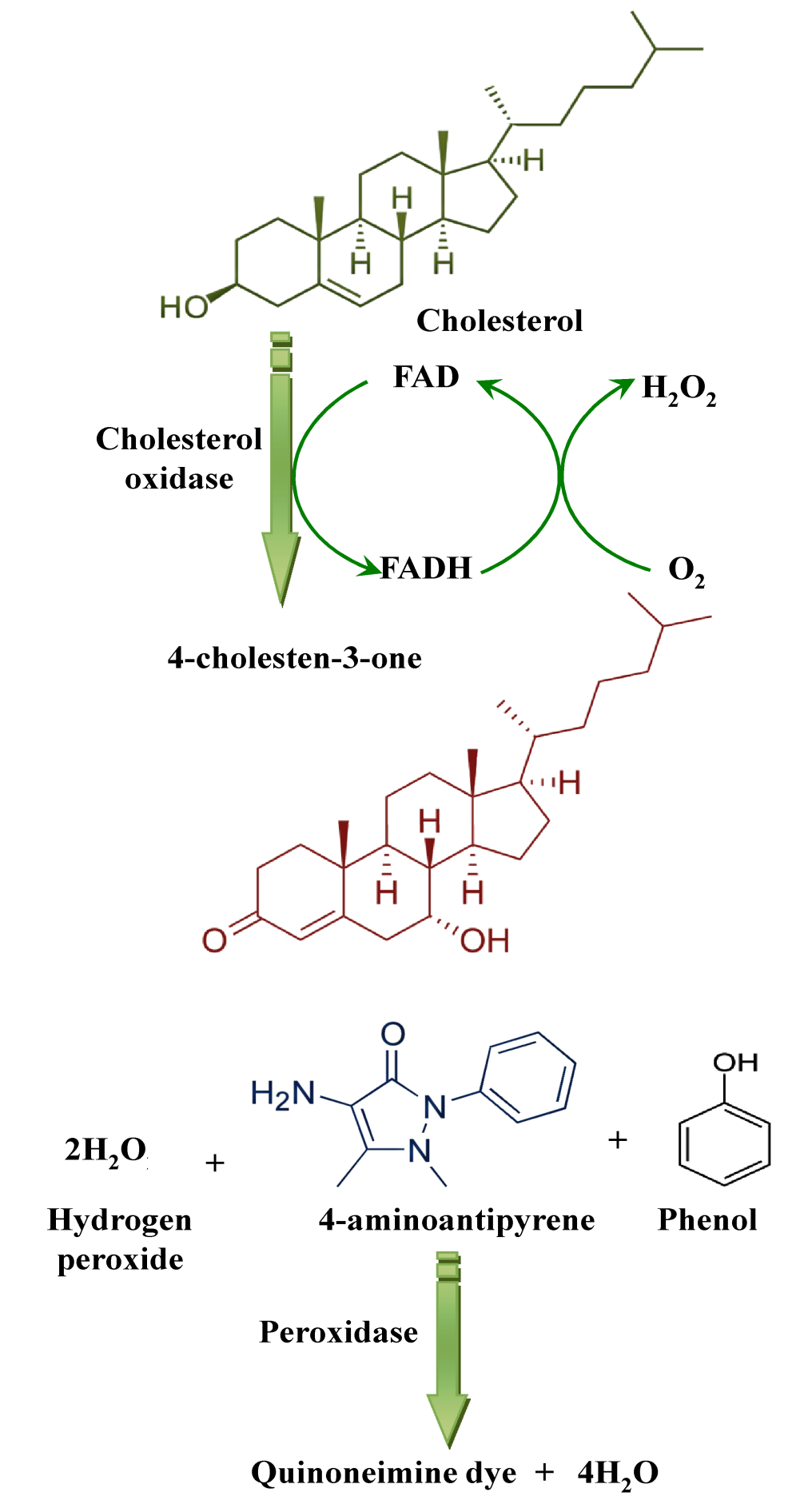
**

**Supplementary Figure 1.** Mechanism of reaction catalyzed by cholesterol oxidase.
